# Supplementary material for: Patterned gastrointestinal monolayers with bilateral access as observable models of parasite gut infection
Source: Nat Biomed Eng. Author manuscript; Available in PMC 2025 Jan 23. (PMC7617323; doi:10.1038/s41551-024-01313-4)
Supplement: Supplementary Information [file EMS202185-supplement-Supplementary_Information.pdf]

nature biomedical engineering

Supplementary information https://doi.org/10.1038/s41551-024-01313-4

Patterned gastrointestinal monolayers with bilateral access as observable models of parasite gut infection

In the format provided by the authors and unedited

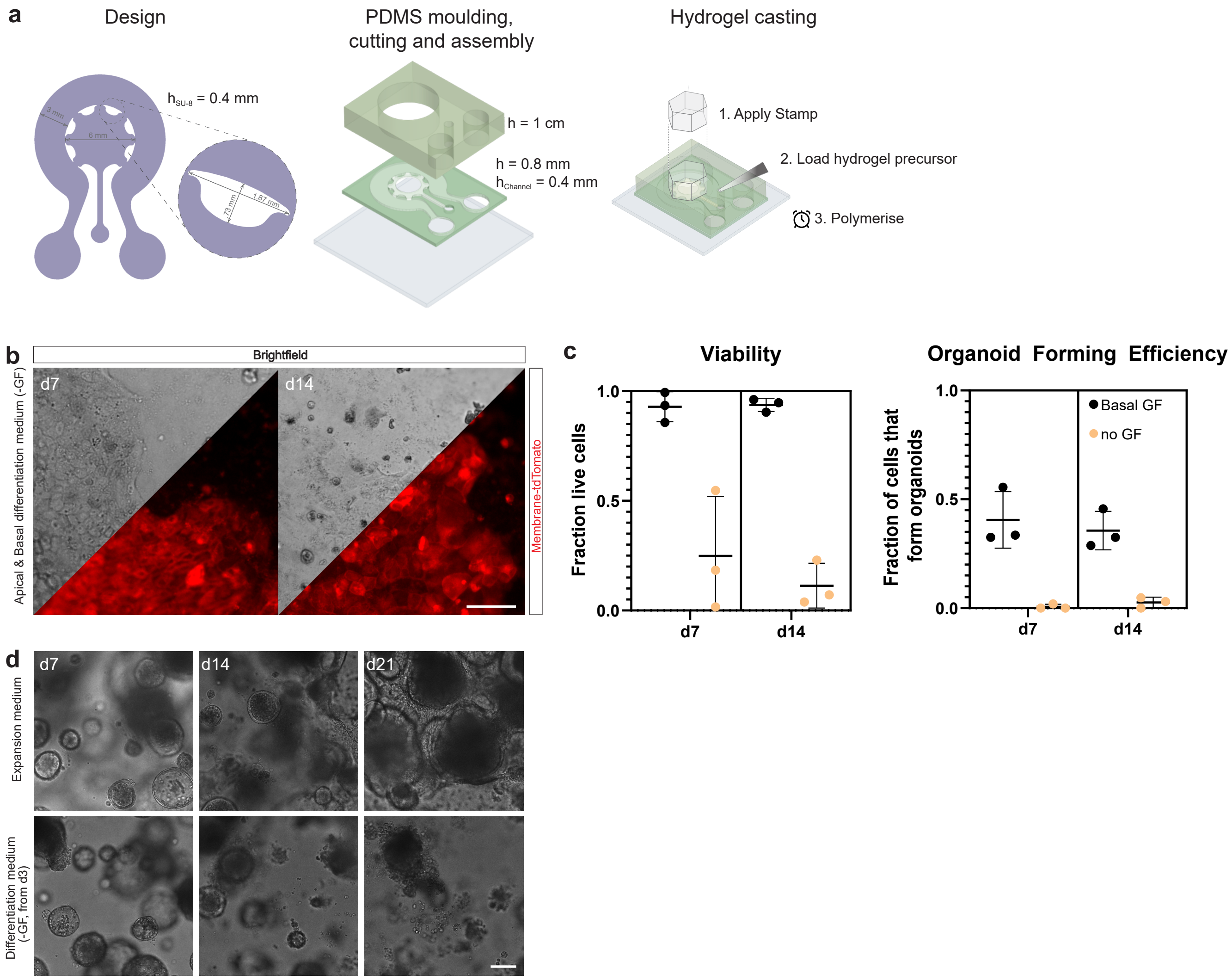

Supplementary Fig. 1 I Fabrication of Transgel device. a, Design and fabrication of Transgel devices. First, a microfabricated wafer with SU-8 topology was prepared, used for the first layer of Transgel device. This layer is oxygen-plasma-bonded to glass, and a well-like superstructure is bonded onto it. To cast the hydrogel, a polydimethylsiloxane (PDMS) stamp is temporarily applied at the opening, and hydrogel precursor solution is loaded via the hydrogel access port. **b,c,** Control to Fig. 1g-i, Brightfield picture (b) and assessment of cell viability and organoid forming efficiency (c) of day 7 and day 14 of gastric epithelial cells when removing of stem cell growth factors (GFs) also from the basal side medium from day 3 on. Epithelium maintenance is impaired, showing that basally delivered GFs are necessary for epithelium maintenance. Representative images of three independent experiments. Cells were collected from Transgel hydrogels and analyzed for viability and capacity to form organoids. Mean and SD from three independent experiments are shown. **d,** Brightfield image sequence of gastric organoids in expansion (with GFs) and differentiation (without GF), related to Fig. 1i. Representative images of three independent experiments. b,d, Scale bars, 100 µm.

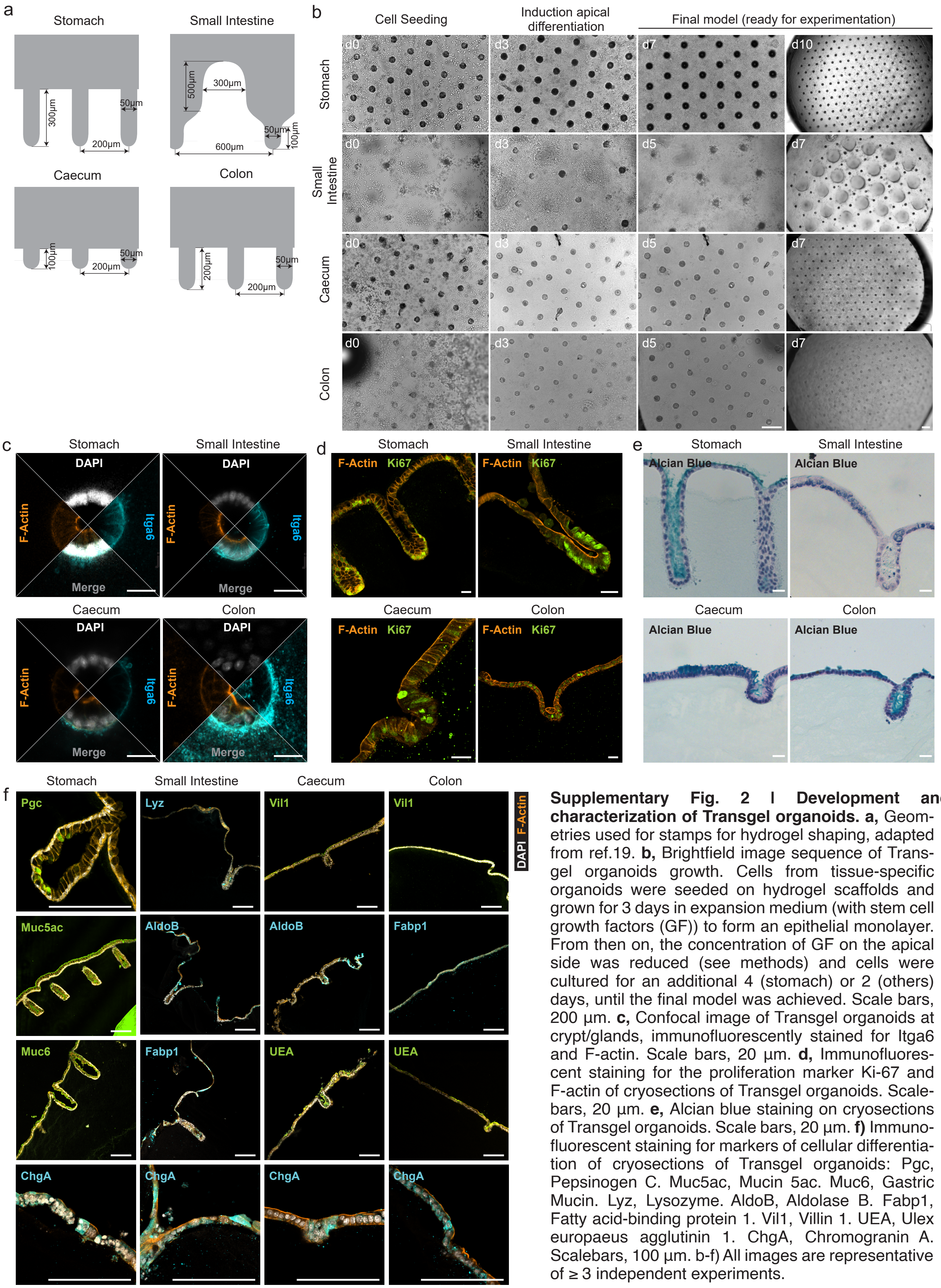

Supplementary Fig. 2 I Development and characterization of Transgel organoids. a, Geometries used for stamps for hydrogel shaping, adapted from ref.19. **b,** Brightfield image sequence of Transgel organoids growth. Cells from tissue-specific organoids were seeded on hydrogel scaffolds and grown for 3 days in expansion medium (with stem cell growth factors (GF)) to form an epithelial monolayer. From then on, the concentration of GF on the apical side was reduced (see methods) and cells were cultured for an additional 4 (stomach) or 2 (others) days, until the final model was achieved. Scale bars, 200 µm. **c,** Confocal image of Transgel organoids at crypt/glands, immunofluorescently stained for Itga6 and F-actin. Scale bars, 20 µm. **d,** Immunofluorescent staining for the proliferation marker Ki-67 and F-actin of cryosections of Transgel organoids. Scalebars, 20 µm. **e,** Alcian blue staining on cryosections of Transgel organoids. Scale bars, 20 µm. **f)** Immunofluorescent staining for markers of cellular differentiation of cryosections of Transgel organoids: Pgc, Pepsinogen C. Muc5ac, Mucin 5ac. Muc6, Gastric Mucin. Lyz, Lysozyme. AldoB, Aldolase B. Fabp1, Fatty acid-binding protein 1. Vil1, Villin 1. UEA, Ulex europaeus agglutinin 1. ChgA, Chromogranin A. Scalebars, 100 µm. b-f) All images are representative of ≥ 3 independent experiments.

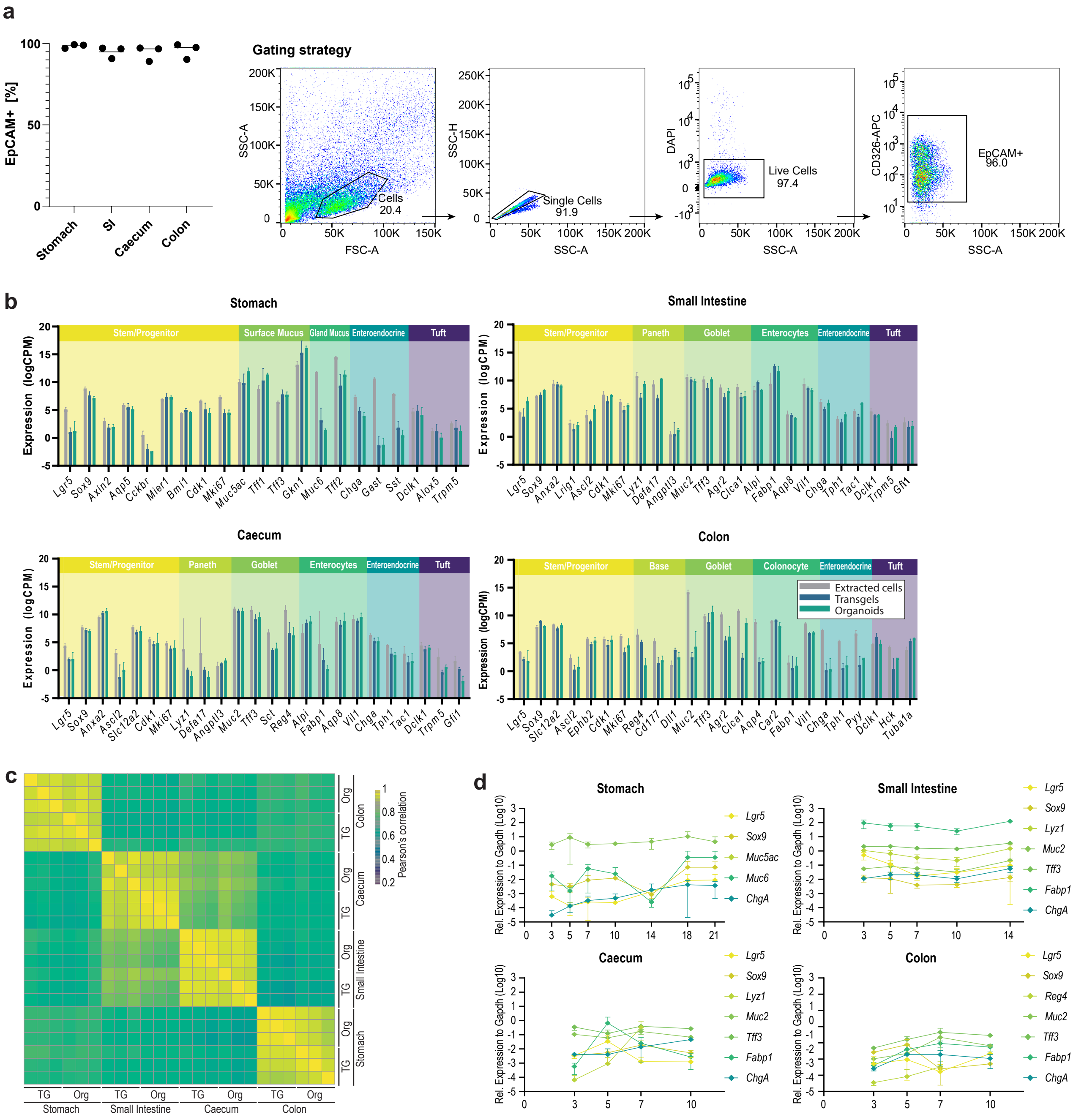

Supplementary Fig. 3 | Transcriptional comparison between organoids grown in 3D and Transgel cultures. a, Purity assessment of freshly extracted epithelial cells used as reference for RNA sequencing experiment by flow cytometry. Fraction of EpCAM+ cells gated for cells, single cells, live cells. Each point represents one independent extraction from a different mouse, horizontal bar represents mean value. **b,** Expression levels (mean and SD of three independent experiments) of genes of interest in Transgel organoids, 3D organoids and freshly extracted epithelial cells. **c,** Correlation score of all in vitro models (Transgel organoids [TG] and 3D organoids [Org]) show high transcriptional correlation between the two culture methods. **d,** Time-course RT-qPCR to follow key markers of cell types over the lifespans of the models.

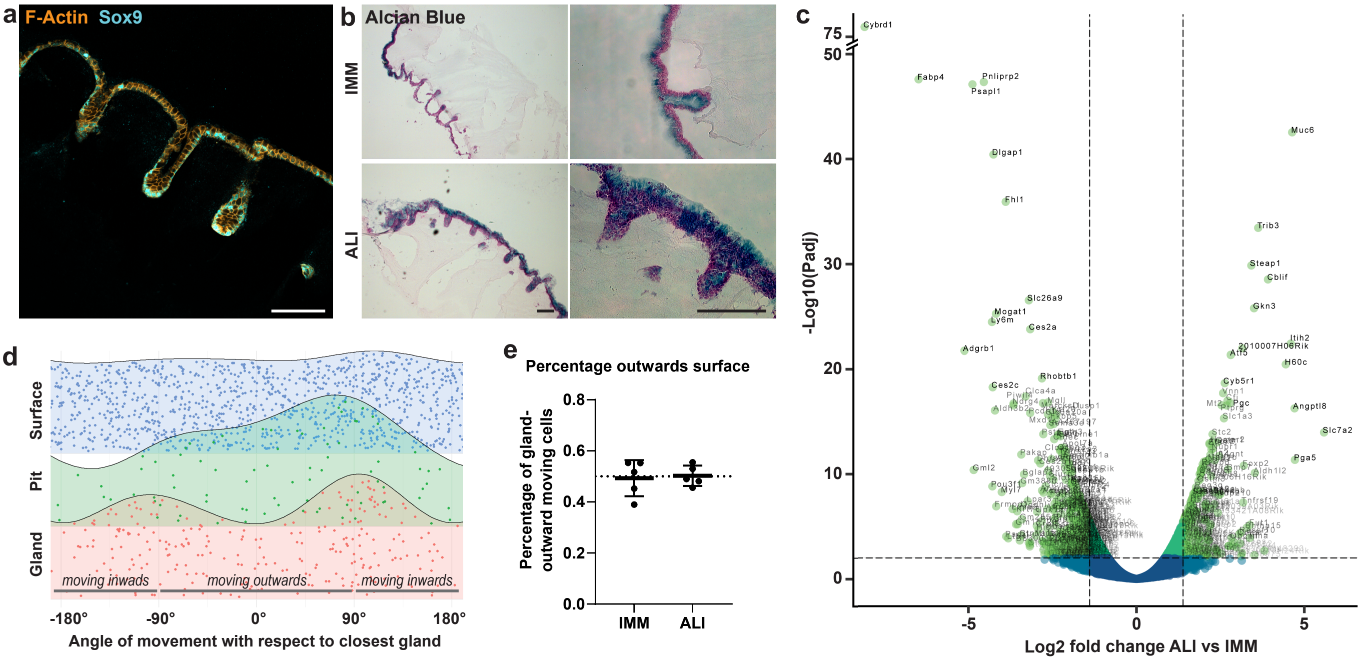

Supplementary Fig. 4 | Characterization and comparison of Transgel organoids cultured in air-liquid interface (ALI) and immersion (IMM) culture. a, Immunofluorescent staining of Sox9 and F-actin of gastric Transgel organoid in ALI culture. Representative images of three independent experiments. Scale bar, 100 µm. **b,** Alcian Blue staining of gastric Transgel organoids in IMM and ALI culture. Scale bars, 100 µm. **c,** Volcano plot of transcriptional comparison between ALI and IMM culture. Related to Fig. 3h. **d,** Representative distribution of angle of movement with respect to closest gland per cell, categorized by location of cell. **e,** Percentage of cells in the surface regions that move outwards from glands. Each point represents one independent experiment and horizontal line represents mean with SD. Dotted horizontal line represents expected value of 50%.

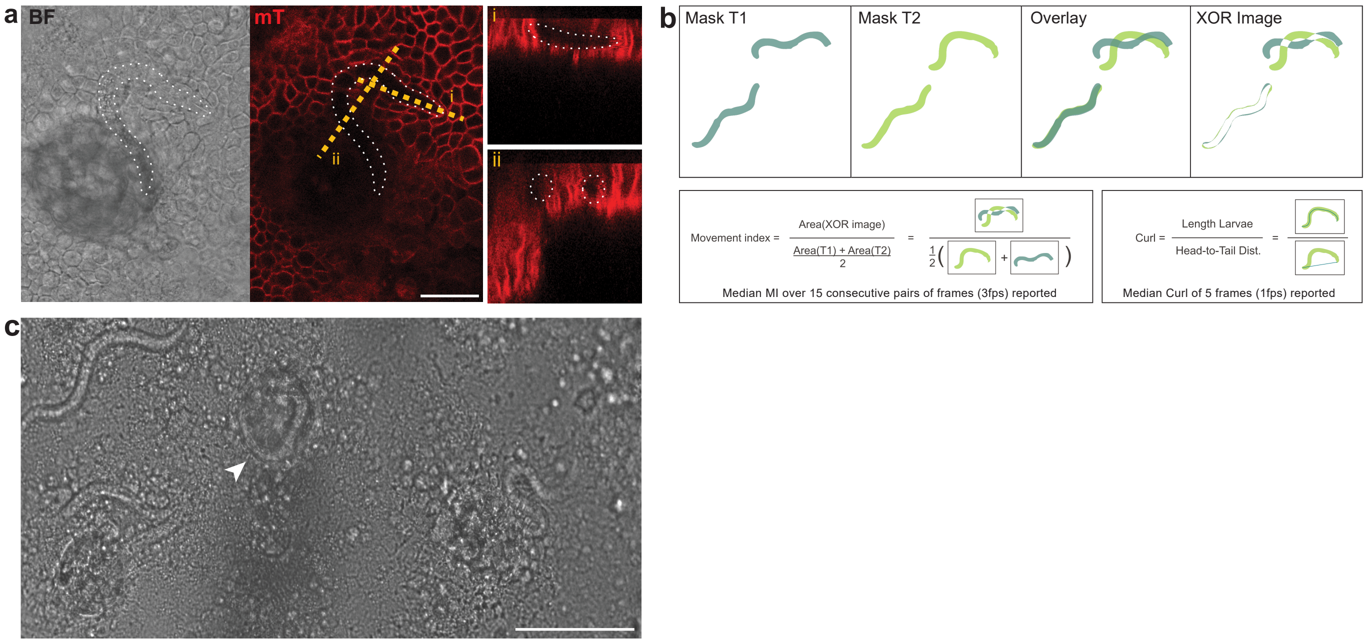

Supplementary Fig. 5 l Extended data to T. muris infection on caecal Transgel organoids. a, Confocal image and side projections (right) of live caecal Transgel organoids with intracellular L1 T. muris larvae. Representative images of four independent experiments. Scale bar, 20 µm. **b,** Analysis strategy of movement index and curl of larvae. Related to Fig. 4d,e. **c,** Example of larvae localized in the crypt region. Scale bar, 100 µm.

**Stomach**		**Small Intestine**		**Caecum**		**Colon**		**Supplier**
Expansion	*Differentiation*	*Expansion*	*Differentiation*	*Expansion*	*Differentiation*	*Expansion*	*Differentiation*	
Adv. DMEM/F-12		Adv. DMEM/F-12		Adv. DMEM/F-12		Adv. DMEM/F-12		Gibco, 12634010
50% L-WRN Conditioned medium	10% L-WRN Conditioned medium			50% L-WRN Conditioned medium	20% L-WRN Conditioned medium	50% L-WRN Conditioned medium	10% L-WRN Conditioned medium	In-house
HEPES, 10mM		HEPES, 10mM		HEPES, 10mM		HEPES, 10mM		Gibco, 15630080
GlutaMAX-I		GlutaMAX-I		GlutaMAX-I		GlutaMAX-I		Gibco, 35050061
B27 supplement		B27 supplement		B27 supplement		B27 supplement		Gibco, 17504044
		N2 supplement		N2 supplement		N2 supplement		Gibco, 12634010
EGF, 50ng/ml	EGF, 5ng/ml	EGF, 50ng/ml		EGF, 50ng/ml		EGF, 50ng/ml		Peprotech, 315-09
FGF-10, 200ng/ml				FGF-10, 100ng/ml				Peprotech, 100-18B
A83-01, 0.5µM						A83-01, 0.5µM		Stemgen, 41730
[Leu15]-Gastrin, 10nM								Sigma-Aldrich; G9145
		Noggin, 0.1µg/ml						EPFL Protein Expression Core Facility
		R-Spondin, 0.5µg/ml						EPFL Protein Expression Core Facility
		CHIR99021, 3µM						STEMCELL, 100-1042
		Valporic Acid, 1mM						STEMCELL, 72292

Supplementary Table 1 l Media formulations for organoid and Transgel organoid culture.

Target	Conjugate	Dilution	Supplier
Primary Antibodies			
Sox9		1:200	Abcam, ab185966
Ki67		1:100	BD Biosciences, 550609
Itga6		1:100	Sigma Aldrich, MAB1378
Muc5ac		1:100	Abcam, ab3649
Muc6		1:500	Abcam, ab212646
Pgc		1:200	Abcam, ab255826
Lyz		1:100	Invitrogen, PA1-29680
AldoB		1:100	Abcam, ab129728
Vil1		1:100	Santa Cruz Biotechnology, sc-58897
Fabp1		1:100	R&D Systems, AF1565
GFP		1:100	Abcam, ab13970
ChgA		1:100	Abcam, ab15160
P43		1:100	Prof Richard Grencis, University of Manchester
CD326 (EpCAM)	APC	1:800 (FC)	Invitrogen, 17-5791-82
Secondary Antibodies			
Mouse IgG	Alexa Fluor 488	1:400	Invitrogen, A-11029
Rabbit IgG	Alexa Fluor 647	1:400	Invitrogen, A-31573
Rat IgG	Alexa Fluor 647	1:400	Invitrogen, A-21247
Goat IgG	Alexa Fluor 488	1:400	Invitrogen, A-11055
Chicken IgY	Alexa Fluor 488	1:400	Abcam, ab150173
Others			
Phalloidin	Alexa Fluor 546	1:100	Invitrogen, A22283
Ulex Europaeus (Gorse) Agglutinin I (UEA I)	Fluorescin (FITC)	1:400	Invitrogen, L32476
DAPI		2.5µg/ml (IF), 0.5µg/ml (FC)	Sigma-Aldrich, D9542

Supplementary Table 2 I Antibodies and Dyes used for Immunofluorescent stainings (IF) and flow cytometry (FC). APC, Allophycocyanin; DAPI, 4′,6-diamidino-2-phenylindole.

Target	FW	REV
Gapdh	ATCCTGCACCACCAACTGCT	GGGCCATCCACAGTCTTCTG
Lgr5	ATTCGGTGCATTTAGCTTGG	CGAACACCTGCGTGAATATG
Sox9	CTCCGGCATGAGTGAGGTG	TCAGTTCACCGATGTCCACG
Fabp1	AGGGGGTGTCAGAAATCGTG	CACCTTCCAGCTTGACGACT
Muc2	TCCTGACCAAGAGCGAACAC	ACAGCACGACAGTCTTCAGG
Muc5ac	TCCCAGACATGTCCCCTCAA	AACATGTGTTGGTGCAGTCAGT
Muc6	GGAACTAACAGTCTGGACCACC	CTTCGGTATGGATGTAGGAGGC
Tff3	CAGAGCCCTCTGGCTAATGC	GGCACCATACATTGGCTTGG
Lyz1	GTCACACTTCCTCGCTTTCC	TGGCTTTGCTGACTGACAAG
Reg4	GTGTGGATTGGCCTGCATGA	AGATCAGCCACCAGGTGAGA
ChgA	CTGTGAACAGCCCCATGACA	GACAGCGAGTCGGAGATGAC

Supplementary Table 3 l Primer sequences used for qPCR analysis.

Supplementary Video 1 l Animated confocal live-acquired stack images of stomach, small intestine, caecum and colon Transgel organoids. Brightfield and membrane-tdTomato. Related to Fig. 2c.

Supplementary Video 2 l Cellular motility of gastric Transgel organoids in immersion and air-liquid interface cultures. Z-coded confocal time lapse images of membrane-tdTomato cells and single slice images at surface and glands as well as 3D reconstruction. Related to Fig. 3j.

Supplementary Video 3 l Infection of *T. muris* larvae on caecal Transgel organoids. Overview timelapse images of first 18h of infection. Magnification movie 12h p.i. (live) of larvae at different stages of infection. Some successfully invaded the epithelial monolayer and became intracellular. Animated live-acquired stack image of intracellular larvae, brightfield and membrane-tdTomato channels. Related to Fig. 4a-e.

Supplementary Video 4 l *Trichuris muris* larvae moving through epithelium by tunnel formation. Live observation of single larvae by brightfield timelapse imaging. Confocal imaging with staining for dead cells (SYTOX Blue) and apoptosis (Casp3). High magnification movie of larval head movements. Related to Fig. 4f-h.
